# A stratified approach to juvenile idiopathic arthritis: leveraging machine learning to uncover subgroups of patients following treatment with etanercept

**DOI:** 10.1093/rap/rkag088

**Published:** 2026-07-20

**Authors:** Stephanie J W Shoop-Worrall, Saskia Lawson-Tovey, UK JIA Biologics Register Investigators, CAPS Investigators, CHARMS Investigators, CLUSTER Consortium, Lucy R Wedderburn, Andrew Dick, Michael W Beresford, Athimalaipet V Ramanan, Michael Barnes, Stephen Eyre, Kimme Hyrich, Soumya Raychaudhuri, Chris Wallace, Nophar Geifman, Lucy R Wedderburn, Melissa Kartawinata, Zoe Wanstall, Bethany R Jebson, Alyssia McNeece, Elizabeth Ralph, Vasiliki Alexiou, Fatjon Dekaj, Aline Kimonyo, Fatema Merali, Emma Sumner, Emily Robinson, Andrew Dick, Michael W Beresford, Emil Carlsson, Joanna Fairlie, Jenna F Gritzfeld, Athimalaipet Ramanan, Teresa Duerr, Michael Barnes, Sandra Ng, Kimme Hyrich, Stephen Eyre, Soumya Raychaudhuri, Andrew Morris, Annie Yarwood, Samantha Smith, Stevie Shoop-Worrall, Saskia Lawson-Tovey, John Bowes, Paul Martin, Melissa Tordoff, Michael Stadler, Wendy Thomson, Damian Tarasek, Chris Wallace, Wei-Yu Lin, Nophar Geifman, Sarah Clarke, Victoria J Burton, Thierry Sornasse, Daniela Dastros-Pitei, Sumanta Mukherjee, Jacqui Roberts, Rami Kallala, Helen Neale, John Ioannou, Hussein Al-Mossawi, Eslam Al-Abadi, Vicky Alexiou, Cherelle Allen, Kate Armon, Rehana Begum, Rumena Begum, Mariejennelynn Bostock, Katrin Buerkle, Charlotte Busby, Maryam Butt, Nga Sze (Emily) Cheng, Chia-Ping Chou, Joanna Cobb, Louise Coke, Julie Cook, Jenny Crook, Serena Cruickshank-Hull, Karen Davies, Lucinda Dawson, Fatjon Dekaj, Monika Dimitrova, Julie Enright, Angela Etheridge, Elizabeth (Lizzie) Fofana, Sara Foster, Sophie Foxall, Paul Gilbert, Genevieve Gottschalk, Eileen Hahn, Jeannette Hall, Daniel Hawley, Anne Hinks, Shashi Hirani, Ruth Howman, Alisha Hussein, Fatema Jeraj, Emma Jordan, Melissa Kartawinata, Laura Kassoumeri, Aline Kimonyo, Klaudia Kupiec, Sham Lal, Alice Leahy, Freya Luling Feilding, Ian MacDonald, Alyssia McNeece, Laura Melville, Fatima Merali, Halima Moncrieffe, Gudrun Moore, Kathleen Mulligan, Stanton Newman, Lucy Nguyen, Fiona Patrick, Hannah Peckham, Chadwick Pils, Elizabeth Ralph, Rachel Rikunenko, Emily Robinson, Jennie Sharp, Taunton Southwood, Jason Sowter, Mohammed Zaffar Ullah, Wendy Thomson, Simona Ursu, Hemlata Varsani, Kishore Warrier, Lucy R Wedderburn, Pamela Whitworth, Rachel Wiffen, Alexis Wormall, Patricia Woo, Lucy R Wedderburn, Kimme L Hyrich, Nophar Geifman

**Affiliations:** Centre for Epidemiology Versus Arthritis, University of Manchester, Manchester, UK; Centre for Health Informatics, University of Manchester, Manchester, UK; Centre for Genetics and Genomics Versus Arthritis, University of Manchester, Manchester, UK; National Institute for Health and Care Research Manchester Biomedical Research Centre, Manchester University Hospitals NHS Foundation Trust, Manchester, UK; Centre for Adolescent Rheumatology Versus Arthritis at UCL, UCLH and GOSH, London, UK; Infection, Immunity and Inflammation Research & Teaching Department, UCL GOS Institute of Child Health, London, UK; Great Ormond Street Hospital Biomedical Research Centre, National Institute for Health and Care Research, London, UK; Centre for Epidemiology Versus Arthritis, University of Manchester, Manchester, UK; National Institute for Health and Care Research Manchester Biomedical Research Centre, Manchester University Hospitals NHS Foundation Trust, Manchester, UK; School of Health Sciences, Faculty of Health and Medical Sciences, University of Surrey, Surrey, UK

**Keywords:** biologics, stratified medicine, juvenile idiopathic arthritis, outcomes, epidemiology

## Abstract

**Objectives:**

To facilitate a stratified approach to treatment of JIA and uncover trajectory-based patterns of key clinical outcomes following etanercept (ETN) initiation in children and young people (CYP).

**Methods:**

ETN-naïve CYP with non-systemic JIA recruited to one of three UK prospective multicentre cohorts were selected if they started ETN between 2001 and 2019. JADAS71 (71-joint Juvenile Arthritis Disease Activity) components [active joint count, physician global assessment (PGA), parent global evaluation (PGE) and ESR] were collected in the year following ETN initiation. Clusters of CYP based on JADAS71 component trajectory patterns following ETN initiation were identified using multivariate group-based trajectory models, adjusting for year of ETN initiation. Multinomial logistic regression identified independent predictors of outcome clusters. In a subset of CYP with previously determined MTX trajectories, response clusters were compared descriptively.

**Results:**

In 1003 CYP, five clusters were identified following ETN initiation. In three clusters, JADAS components changed in parallel: fast improvers (16%), slow improvers (10%) and persistent disease (37%). In two, all components except for one improved over time: persistent PGA (7%) and persistent PGE (30%). Where both MTX and ETN trajectories were available (*n* = 139), 69% of CYP with persistent disease following MTX were assigned to other response clusters following ETN initiation. There was high consistency (73%) of CYP having a persistent PGE pattern following both drugs.

**Conclusion:**

Across multiple DMARDs, JIA disease activity does not always improve to similar degrees across the different features of disease. A majority of CYP initially experiencing persistent disease with MTX fall into a response group following ETN.

Key messages

- Clinical trial outcomes may miss heterogeneity of response across individual disease features of JIA.
- Stratified treatment requires an understanding of which disease features respond to therapy in which individuals.
- Verifiable clusters of JIA have systematically different disease patterns across disease features following ETN.

## Introduction

Currently in clinical research of pharmaceuticals used to treat rheumatic diseases, including clinical trials and analyses of observational data, measurement of response is usually defined using a binary outcome of response or non-response measured at a single point in time and defined using composite outcome measures. In JIA clinical trials, this is often determined using the ACR Pedi scores [1], while more recently, improvement based on Juvenile Arthritis Disease Activity (JADAS) cut-points has also been incorporated [2]. This has an advantage of giving an overall measure at the population level of expected response based on this composite outcome but then assumes that all response is the ‘same’. By categorising response using a single time-point binary composite paradigm, the opportunity to understand how well treatment affects individual aspects of disease is lost, and therefore heterogeneity of disease outcome, both within an individual and between individuals is not possible. To better assess drug response in JIA, more granular measures incorporating longitudinal data are needed. These may allow for the identification of differences in improvement among different features of disease with the ultimate aim of identifying and characterising groups of children whose disease will respond differently to therapies, furthering a stratified medicine approach in this disease.

Previously, using unsupervised machine learning methods, clusters with different disease outcome trajectories following MTX initiation were identified based on the four core outcome variables contained within the JADAS score and verified in independent cohorts [3]. Clusters differed in terms of their degree and rate of improvement across the four individual components of the JADAS. These clusters demonstrate an initial step toward characterising personalised response measures in JIA. However, it is unclear whether such groupings of response across the individual JADAS components are unique to MTX or common and seen following other anti-rheumatic therapies. Etanercept is one of the most commonly prescribed first-line biologic DMARDs for JIA [4]. This study aimed to understand trajectories of disease outcomes in children and young people (CYP) with JIA across the UK initiating this drug for the first time.

## Methods

### Study population

The CLUSTER consortium includes three prospective, multicentre, UK observational cohorts following CYP with JIA: the Childhood Arthritis Prospective Study (CAPS), the UK JIA Biologics Register [consisting of two parallel studies: the British Society for Paediatric and Adolescent Rheumatology Etanercept Cohort Study (BSPAR-ETN) and Biologics for Children with Rheumatic Diseases (BCRD)] and the Childhood Arthritis Response to Medication Study (CHARMS). These cohorts within CLUSTER have been described previously [3]. All cohorts include and follow CYP who are ETN-naïve and starting ETN. All studies had NHS Health Research Authority approval and written informed consent was provided by guardians or participants with assent additionally provided by participants where appropriate.

CYP were included in the current analysis if they had a physician’s diagnosis of non-systemic JIA and initiated ETN prior to 1 January 2019, to allow for at least 1 year of follow-up at the time of analysis. Those with no recorded ETN start date and those who were recorded to have no active joints or were indicated to have clinically inactive disease according to Wallace preliminary criteria [5] or JADAS cut-offs [6] at the start of ETN were excluded. In addition, those with no data on all four JADAS components in a single follow-up over the year after starting ETN were excluded.

### Data collection

CAPS collects data from initial presentation to paediatric rheumatology and annually thereafter. Between 2001 and 2010, a 6-month follow-up was additionally included. There were no ETN-specific follow-up time points. For the other studies, baseline was defined at initiation of ETN and these studies include a 3-month (BSPAR-ETN prior to 2008), 6-month (BSPAR-ETN, BCRD, CHARMS) and 12-month follow-up (BSPAR-ETN, BCRD). At each follow-up, demographic and clinical information, including medication data, were extracted from electronic patient records by study nurses and participants/guardians completed patient-reported questionnaires, including the Childhood Health Assessment Questionnaire (CHAQ), which incorporates two 100-mm visual analogue scales: pain and a parent global evaluation of well-being.

Although each study had predefined time points for data collection, date of actual data collection mapped to routine clinic visits and exact dates, rounded to the nearest month, were used in analysis. Data collected within 3 months prior to ETN initiation were used as baseline and reset to the ETN start date. Any data more than 3 months before ETN initiation were excluded. Follow-up was extended to 14 months to allow for variable data collection at the 12-month follow-up. If CYP had been recruited to multiple studies within the CLUSTER consortium, all follow-up points within the 14-month period were included, even if from different studies.

### Outcome measures

The primary outcome measures were the four components of the 71-joint JADAS (JADAS71): active joint count (AJC), physician’s global assessment of disease activity (PGA; 0.0–10.0 cm), parent global evaluation of well-being (PGE; 0.0–10.0 cm) and ESR (mm/h).

### Statistical analysis

#### Multivariate trajectory modelling

Multivariate group-based trajectory models were used to identify clusters of CYP with different longitudinal patterns across the four JADAS components over the year following ETN initiation. The models were tested for 1–10 groups/clusters, with either linear, quadratic or cubic polynomial trajectory shapes [7]. For each model, posterior probabilities for cluster assignment are produced for each participant; participants’ final cluster assignment was based on the highest probability. Outcomes were log1p transformed for analysis and were modelled using censored-normal models. In the early 2000s, ETN was the only licensed biologic therapy for JIA, with further biologics becoming available in the mid- to late 2000s and early 2010s [8]. The choice of available biologics may have confounded individual trajectories, so the primary analysis adjusted for the year of ETN initiation (<2006, ≥2006).

When selecting the optimal model, those resulting with a cluster accounting for <1% of the entire cohort, mean posterior probability for cluster assignment <70% or relative entropy <0.5 were excluded. Model fit (using the Bayesian information criterion), graphical analysis of individual trajectories, clinical plausibility and parsimony were then applied to select the final model [7].

#### Sensitivity analyses

A sensitivity analysis was carried out by adjusting the model for concurrent MTX therapy and a second tested the model unadjusted for both concurrent MTX therapy and year of ETN initiation.

#### Competing risks

This study sought to understand patterns of disease change after ETN initiation, not other biologic therapies. If CYP were switched to alternative biologic therapies (with the exception of an ETN biosimilar), data were censored on the day after the switch to avoid capturing disease changes following initiation of a different biologic DMARD. Any outcome data collected on the day of decision to switch were included since any new therapy would not have affected these outcomes. Additional secondary analyses assessed differences in time to stopping ETN for any reason, and time-to-biologic-switching, within 1 year of ETN initiation among the identified clusters, using univariable Cox regression and Kaplan–Meier plots. Outcomes following ETN cessation where no new therapies were initiated were retained in trajectory models in order to capture the natural disease course following ETN initiation, including remission off medication.

#### Clinical characteristics of ETN trajectory clusters

Demographic and clinical factors collected at ETN initiation were assessed for association with ETN trajectory clusters using univariable and multivariable multinomial logistic regression models both against the ‘worst’ cluster and against all other clusters. Multivariable models included age and disease duration at ETN initiation, gender, ethnicity (categorised into white ethnicity yes/no), ILAR category, the six JIA core outcome variables (AJC, limited joint count, PGA, PGE, CHAQ, ESR), pain and whether MTX was taken concurrently alongside ETN. Collinear variables with lesser clinical relevance were excluded. Models were tested for internal validity using receiver operating characteristics.

#### Missing data

As in maximum likelihood–based methods, group-based trajectory modelling is robust to missing outcome data, provided they are missing at random. Therefore, no imputation of missing data were undertaken for the primary analysis. However, missing data in risk factors included in regression models were imputed via multiple imputation by chained equations over 20 datasets using baseline (ETN initiation) and 6-month data.

All analyses were completed in Stata version 14.0 (StataCorp, College Station, TX, USA). Data visualisation was completed in R version 3.6.1 using RStudio version 1.2.5001 (R Foundation for Statistical Computing, Vienna, Austria).

#### Mapping MTX and ETN trajectories

A prior analysis included 1898 CYP starting MTX for the first time [3]. For the MTX analysis, children were split into discovery (UK JIA Biologics Register) and verification (CAPS, CHARMS) cohorts. Trajectory patterns were verified across the MTX cohorts (fast improver, slow improver, persistent PGA/PGE, persistent disease). The initially identified ‘relapse’ group was not verified, largely due to limited follow-up time points in the verification cohorts. In order to streamline the comparison of MTX and ETN trajectory cluster comparison, for the current analysis, MTX data were pooled and trajectory analyses rerun, consolidating CYP into one of five MTX trajectory groups as previously discussed: fast improvers, slow improvers, persistent PGA, persistent PGE and persistent disease.

For this additional analysis, CYP who had previously been assigned an MTX trajectory group were retained for the current comparison if they then went on to initiate ETN and had an assigned ETN trajectory group. Mapping between trajectory group assignments was explored descriptively and via chord diagrams.

#### Patient and public involvement

This study involved the CLUSTER Champions patient stakeholder group in its premise, design, methods, interpretation and interim dissemination materials co-production. The Your Rheum group was also involved in the study design and results interpretation.

## Results

### Study population

A total of 1534 CYP had initiated ETN and were recruited to at least one of CAPS (*n* = 5), Biologics Register (*n* = 1118) or CHARMS (*n* = 71) prior to January 2019. Of those, for 154 children the JADAS components provided at the time of ETN initiation indicated clinically inactive disease, 368 had no JADAS data available for the study period and 9 did not have JIA, leaving 1003 CYP for analysis. A total of 163 (16%) CYP initiated ETN prior to 2006 and 501 (50%) were receiving concurrent MTX at the start of ETN. Those excluded from analyses were similar in demographic features, patient-reported outcomes such as CHAQ and pain, and the majority of clinical variables.

The median age was 12 years [interquartile range (IQR) 8–14] and the majority were female (69%), of white ethnicity (90%) and the most common ILAR category was RF-negative polyarticular JIA (37%) (Supplementary Table S1). The three cohorts had largely similar characteristics (Supplementary Table S1).

### Five trajectories of disease impact following ETN initiation identified

**Figure 1:**
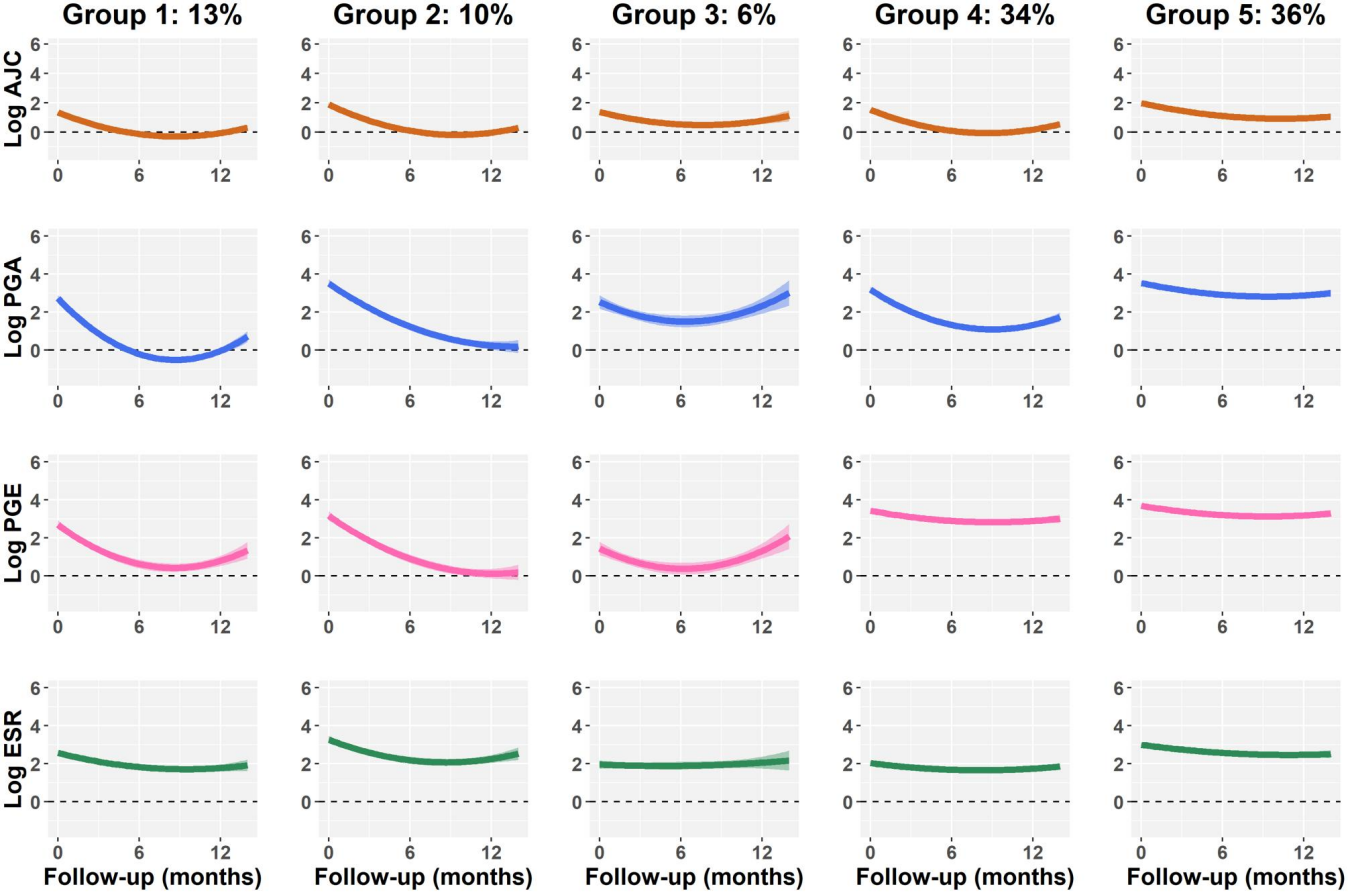
Average log1p transformed active joint count, physician global, patient/parent global and ESR trajectories within five multivariate disease clusters over the year following ETN initiation. Shaded areas represent 95% CIs around fitted mean trajectories

The optimal model resulted in five quadratic trajectory clusters (Fig. 1, Supplementary Table S2, Supplementary Figs S1 and S2): fast improvers (16%) and slow improvers (10%) both improved across active joints at similar rates, while global scores improved to 0 cm by ≈6 or 12 months, respectively. Persistent PGA (7%) and persistent PGE (30%) groups showed improvement in AJC and one global score (PGE or PGA, respectively), with the other global score remaining elevated compared with other measures over the period of follow-up. A final persistent disease group (37%) had persistent elevated scores across all measures (Fig. 1) that did not substantially improve over time.

The inclusion of concurrent MTX within the model did not affect the number of resulting clusters or the nature (trajectory shapes) of these. However, the unadjusted model, which did not include concurrent MTX or year of ETN start, resulted in fewer clusters and did not delineate between the persistent PGA and persistent PGE clusters (data not shown).

### Time to stopping ETN or switching to a second biologic

A total of 109 CYP stopped ETN within 1 year of ETN initiation (10.9%), with 61 of these switching to a second biologic (56.0%). Time to stopping ETN for any reason was significantly shorter for those in the persistent disease trajectory cluster compared with the fast improver group [hazard ratio (HR) 0.5 (95% CI 0.2, 1.0)]. There were no other significant differences in time to all-cause stopping between the persistent disease cluster and other clusters (Supplementary Fig. S3a). When analysing those who switched to a second biologic, compared with the persistent disease group, time to switching to a second biologic therapy was significantly longer in the fast improver [HR 0.3 (95% CI 0.1, 0.8)], slow improver [HR 0.1 (95% CI <0.1, 0.7)] and persistent PGE [HR 0.5 (95% CI 0.3, 0.9)] clusters. There was no significant difference in time to switching to a second biologic between the persistent disease and persistent PGA clusters (*P* = 0.339) (Supplementary Fig. S3b).

### Trajectory clusters associate with differing clinical characteristics

**Figure 2:**
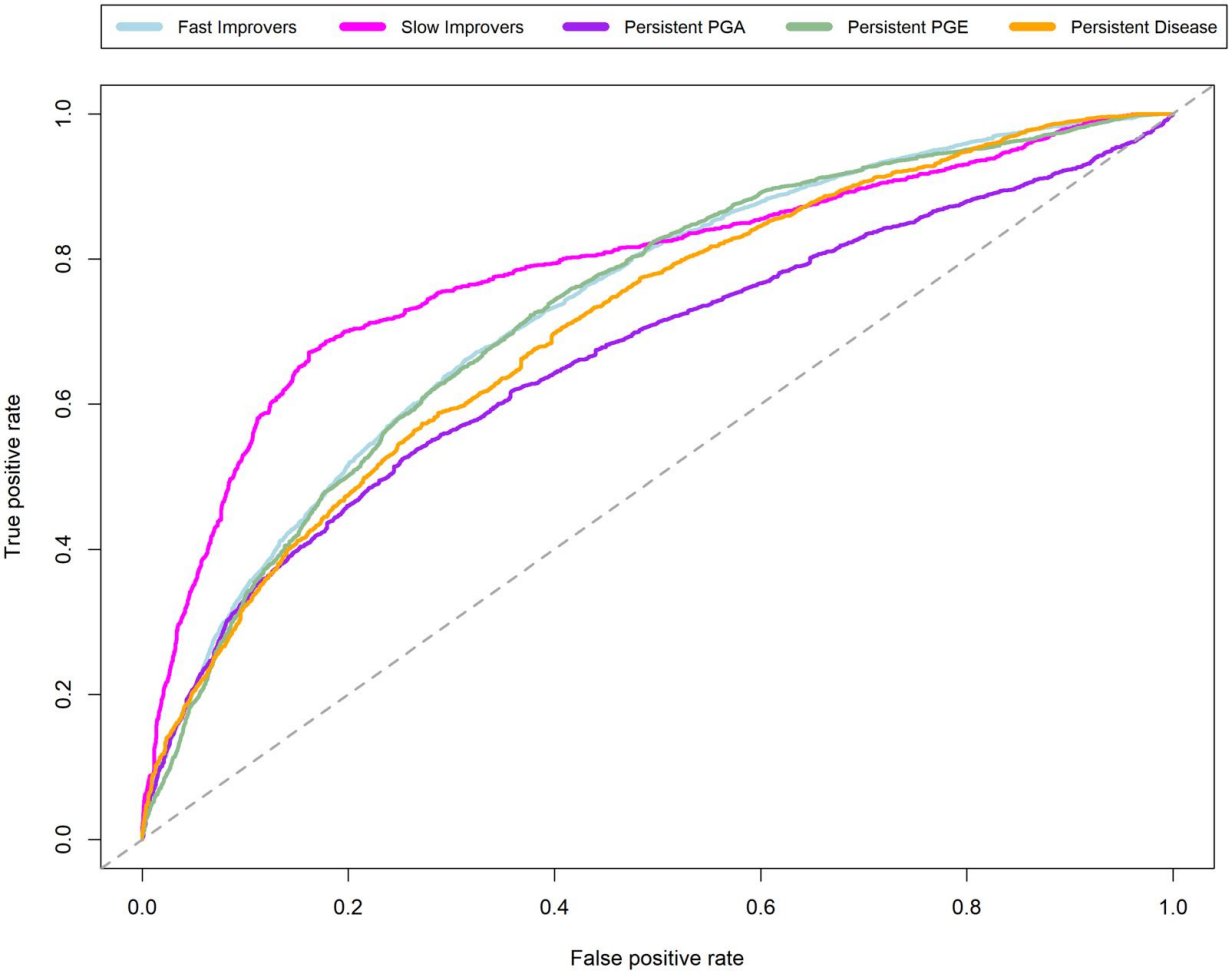
Receiver operating characteristics curves and area under the curve values for predicting ETN improvement cluster membership compared with all other clusters

**Figure 3:**
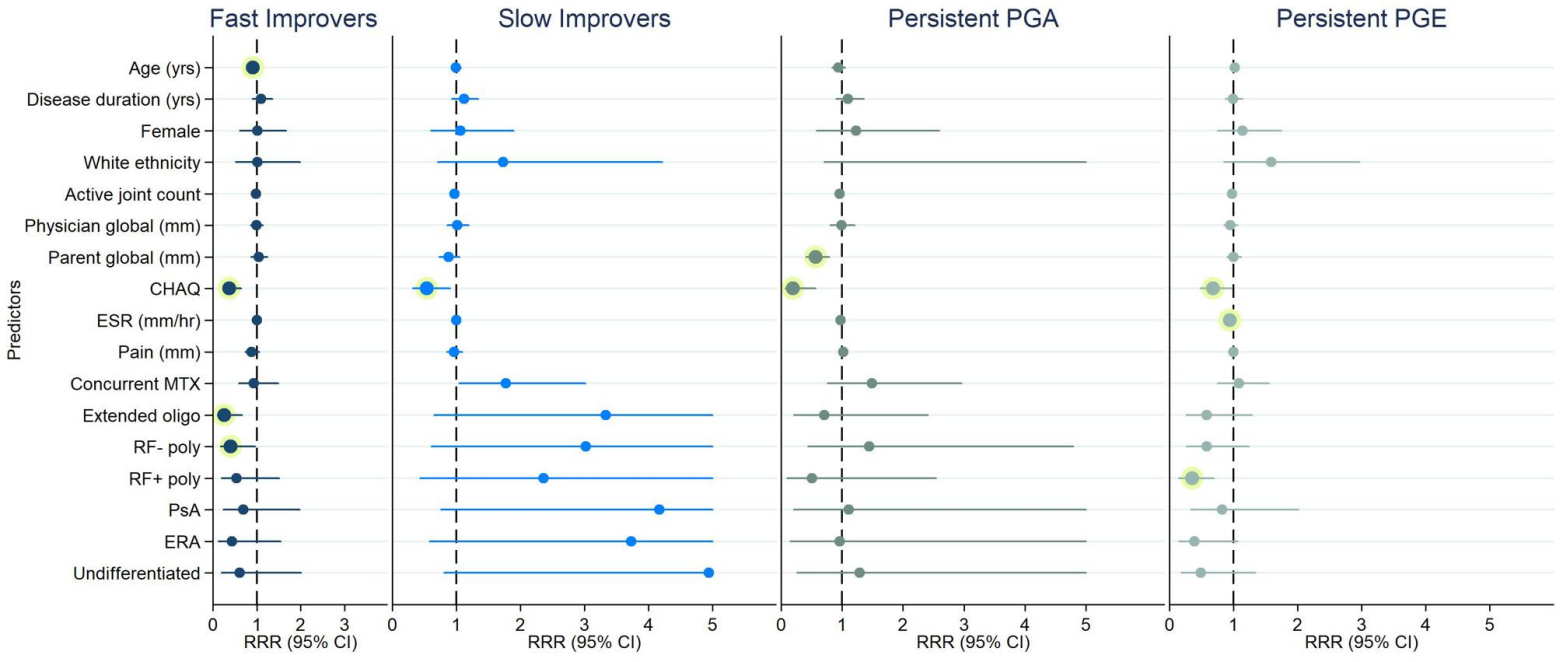
Predictors of disease trajectories following ETN initiation compared with having ‘persistent disease’. Significantly associated variables are highlighted and with figure points. Reference for ILAR category is persistent oligoarthritis. RRR: relative risk ratio

A multivariable regression model developed to identify factors associated with trajectory cluster membership, based on baseline data only (see Methods), resulted in moderate to high area under the receiver operating characteristics curve values for predicting each cluster membership versus all other clusters: fast improvers: 0.77, slow improvers: 0.87, persistent PGA: 0.69, persistent PGE: 0.77 and persistent disease: 0.71 (Fig. 2). Many of the demographic and clinical variables significantly and independently differentiated between the identified trajectory clusters. In particular, age, ILAR category, PGE, CHAQ and ESR scores at ETN initiation, as well as concurrent MTX treatment, were significantly different across the clusters (Supplementary Tables S3–S5, Fig. 3).

### Mapping between trajectories following MTX and ETN

**Figure 4:**
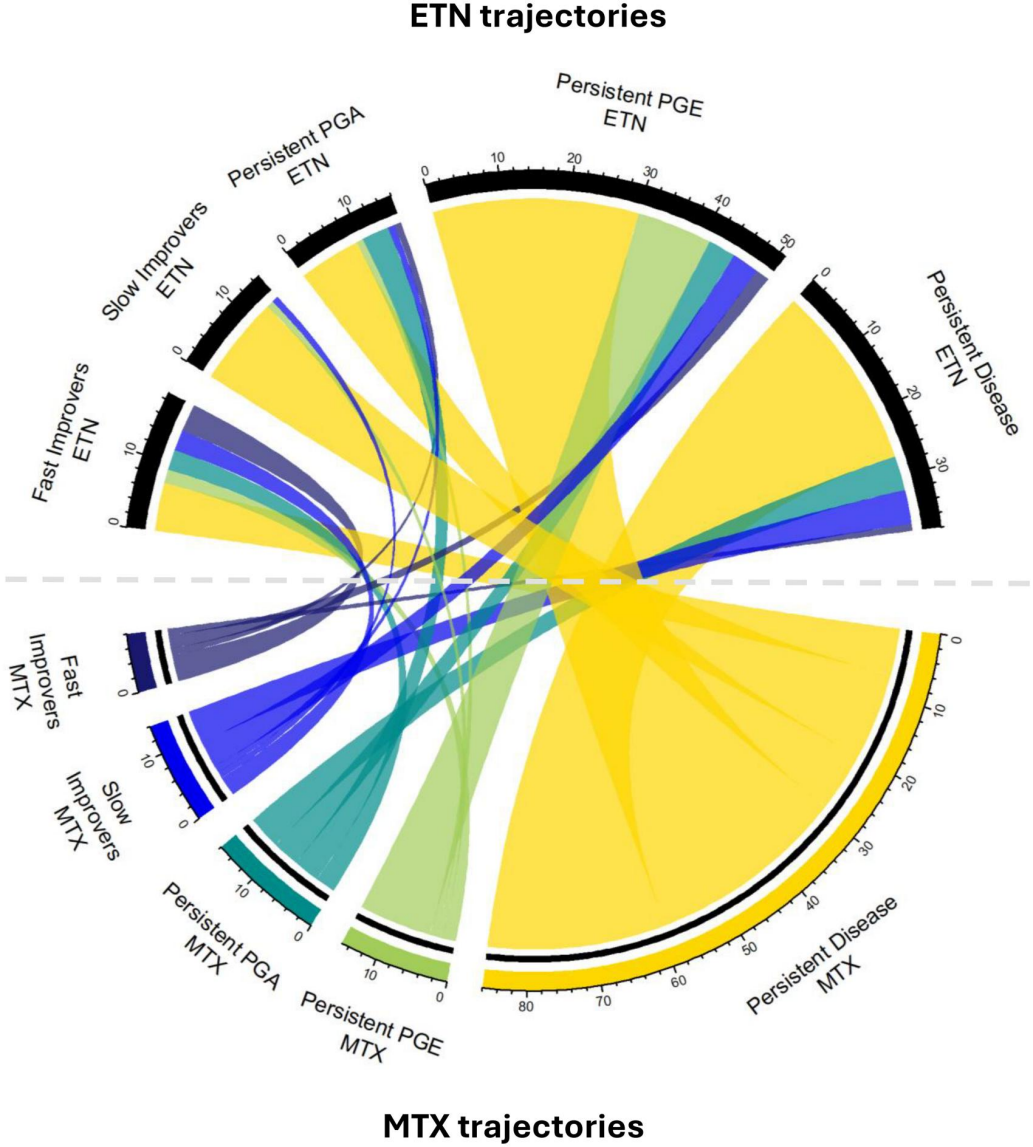
Mapping between MTX trajectories (lower) and ETN trajectories (upper) in CYP who initiated MTX and then ETN between 2001 and 2019

Of 1898 CYP previously used to identify trajectories following MTX initiation [3], 139 had also initiated ETN within the study period, included in the current study, and therefore had been assigned to both MTX and ETN trajectories. Those who experienced ‘persistent PGE’ trajectories following MTX were often clustered into the same trajectory following ETN initiation (73%). However, outside of this pattern, CYP did not frequently fall into the ‘equivalent’ trajectory pattern following both MTX and ETN initiation. Most children (69%) initially categorised into ‘persistent disease’ following MTX initiation were then categorised into fast improvers (8%), slow improvers (15%), persistent PGA (11%) and persistent PGE (35%) following ETN initiation (Fig. 4, Supplementary Table S6).

## Discussion

The current study identified five clusters of JIA with different trajectories of disease outcome following ETN initiation, based on individual components of the JADAS modelled simultaneously in a large population of >1000 CYP. Clusters differed in terms of their degree of concurrence between improvement in physician-reported and parent-reported outcomes over time and overall rates to outcome improvement. Further, several baseline demographic and clinical factors were associated with each of the clusters.

There were noted consistencies between the ETN trajectories identified in the current study and trajectories of disease activity following MTX treatment identified and published previously [3]; largely, the same trajectories were identified. Despite this being a different drug used at a different stage of disease, these data suggest that these are common outcome trajectories following drug initiation for JIA. While similar ‘global’ trajectories are identified following each drug initiation, a sensitivity analysis of those CYP who initiated ETN following treatment with MTX showed that CYP will likely move between clusters. Therefore, a stratified approach to treating JIA could aim for an initial DMARD that will result in CYP experiencing an ‘optimal’ or preferable disease trajectory following its initiation.

Alternative clustering approaches have demonstrated multiple trajectories of disease impact both following initial presentation to paediatric rheumatology [9–11] and following biologic therapy initiation [12] and consistently demonstrate the presence of clusters where improvement occurs at different rates over time [10, 13–17]. Improvement can occur at different rates—over different outcome measures—following treatment, presenting a challenge in assessing treatment response for both clinical trials and practice. In a trial setting, treatment response is generally assessed by a primary endpoint at a specific time point and a decision made as to whether the disease has ‘responded’ to therapies within this time frame. The identification of different rates of improvement suggests a risk of misclassifying ‘responders’ as ‘non-responders’ where the level of response (e.g. ACR Pedi 90) will not be reached until a later time point. This differential misclassification may lead to drugs erroneously being labelled as ineffective in an individual when a longer follow-up may have shown otherwise. Of note, the current ‘fast’ versus ‘slow’ disease improvement groups may or may not represent differences in biological ‘response’ to ETN, or to MTX in the prior analysis [3]. Rather, this trend could be driven by concurrent or earlier steroid or other treatments alongside ETN/MTX, or treatment interruptions (for whatever reason), which are difficult to capture in observational data. The longer-term impacts of this variability in response times needs further exploration, such as potential impact on joint damage, for example. Despite rapid improvements in active joint count across both ‘fast’ and ‘slow’ improvement groups, the main variables showing differences in speed of improvement were physician and parent global scores. This suggests a consistently rapid improvement in inflammatory signs following ETN treatment, but with slower improvements in outward manifestations and impacts of disease in some children. A fast resolution in joint inflammatory activity may therefore, in these instances, not indicate an immediate switch to another biologic.

Several demographic, patient-reported and clinical factors were associated with disease trajectories from the time of ETN initiation and were similar to predictors of MTX clusters previously identified [3]. Younger age and persistent oligoarticular JIA have been associated with optimal response (here, the fast improvers) to multiple DMARDs including ETN, but also with additional outcomes including greater odds of remission across cohorts [9, 18–20]. However, associations were evident beyond ILAR category, allowing for prediction of within-category heterogeneity. Lower initial PGE scores in the persistent PGA group and lower initial ESR in the persistent PGE groups suggest a disparity between physician, lab and parent-/patient-reported outcomes at DMARD initiation may be predictive of future disease course, with the potential for the elevated feature to persist. This was evident where a large proportion of CYP with persistent symptoms (persistent PGE group) following MTX initiation also experienced this disease pattern following ETN. It is unknown whether the children in this cohort received additional interventions to target those factors influencing their well-being scores alongside their ETN treatment, but this finding identifies potential targets for allied healthcare where not currently involved. On the contrary, most children were clustered into a different and arguably more favourable outcome on ETN following MTX. These CYP experienced a range of improvements across the trajectory patterns, again showing variability in response patterns even in a cohort of children who had already experienced treatment with MTX, an important consideration when classifying response to different treatments for JIA.

In contrast to the MTX clusters, lower CHAQ scores at ETN initiation were associated with every trajectory cluster compared with the persistent disease cluster, suggesting greater importance of function in improvements following ETN than MTX. This may also reflect a change in what functional disability reflects in newer-onset JIA versus longer-standing disease. The longer active JIA progresses, the higher risk of both articular and extra-articular damage [21, 22]. Therefore, these findings may reflect a lower propensity to ‘respond’ to treatment in CYP whose disease has gone uncontrolled and led to such damage. This corroborates existing work in the discovery cohort, where traditional ‘response’ to ETN was associated with CHAQ scores in univariable analysis; however, not after adjusting for disease duration [19]. Further work is needed to understand what functional disability scores represent in CYP with shorter and longer-standing disease, including both qualitative and imaging studies. However, this could represent further evidence for the ‘window of opportunity’, where treating early, before the accrual of inflammation-related damage relates to more positive outcome [22].

This study benefitted from the availability of data integrated from three large studies of JIA from across the UK, providing a wealth of information on both treatment outcomes over time and potential predictors of trajectories following treatment, including demographic, clinical and psychosocial factors. The large longitudinal aspects of these cohorts and the ability to link between cohorts also meant that for a smaller cohort, trajectories could be determined and compared across both ETN and MTX. This allowed for the robustness of identified trajectories to be assessed and confirmed over multiple cohorts, drugs and sensitivity analyses. Further, the use of unsupervised machine learning approaches allowed for the identification of clusters of disease that may not be identifiable using a consensus-based (rather than data-driven) approach and provide a greater level of granularity in describing outcomes than the traditional binary response classification.

Limitations of this study include the relatively limited number of data points over time/child due to study design, with a median of 3 time points per child (IQR 2–4). The 1-year visit was only explicitly captured in the UK JIA Biologics Register, with the verification datasets capturing 6 months (CAPS, CHARMS) and usual follow-up annually from diagnosis (CAPS) included if it fell within the 1-year post-MTX-initiation time frame. While the machine learning approach taken here can handle a degree of missing and sparsity in data, greater data capture might improve the granularity of these trajectory patterns further. In particular, flares on a monthly or even weekly basis may not be captured in the current trajectories. Although patient-reported disease impact measures may be able to be collected on such a regular basis through novel digital collection tools [23], gaining clinical measures of disease activity on such a regular basis would likely be unfeasible in terms of cost and burden for both families and healthcare professionals. In addition, the mapping of MTX to ETN trajectories was only possible for those who had taken both drugs within the study window. Those who had initial good response to MTX were underrepresented in the ETN cohort and it is therefore unclear how these CYP would fare following ETN treatment. However, this study focuses on those who needed a biologic therapy in real-world practice and is therefore reflective of the population receiving ETN in the wider JIA population. Finally, the study population was of majority white ethnicity, so the findings may not generalise to more diverse populations.

## Clinical implications

Treatment response in JIA may not be uniform across disease domains and improvement in joint inflammation does not necessarily occur in parallel with improvements in other signs and/or symptoms. This raises a number of clinical implications. First, treatment decisions should consider individual elements of disease and not rely on a single composite score to justify treatment escalation or switching. This could avoid unnecessary switching and/or missing individual discordant features that require intervention. Second, trials of therapies in JIA should include multiple outcomes across the signs and symptoms of disease to capture nuances, population heterogeneity and discordance in response. This could avoid discounting therapies that are highly effective for controlling limited aspects of disease. Third, considering patient- and physician-reported outcomes separately could allow for the identification of children who may benefit from targeted multidisciplinary input, including psychology, physiotherapy or pain management alongside pharmacological therapy.

The identification of ‘fast improver’ and ‘slow improver’ patterns supports a novel, stratified approach within treat-to-target frameworks. While rapid escalation remains appropriate for true non-response, some children may achieve full response with additional time on therapy. Distinguishing these groups is important to avoid premature switching and cycling through limited available treatments, although the consequences of any prolonged time with active inflammation also need to be fully understood and considered.

Finally, these trajectory patterns provide a foundation for future predictive tools to identify likely response profiles at treatment initiation. This has the potential to inform earlier stratification of therapy, optimise treatment and improve long-term outcomes for CYP with JIA. While almost identical disease patterns were observed following the initiation of MTX and ETN, there was considerable shifting from one type of ‘response pattern’ to another between drugs. If future studies provide evidence that such trajectories are predictable using clinical data at treatment initiation, then this opens the door to selection of therapies based on which option is likely to induce the most favourable disease pattern. Future work should also focus on identifying children likely to demonstrate persistent non-response across multiple therapies and may therefore require early access to targeted and novel interventions.

## Conclusions

In CYP starting ETN, five trajectory patterns of JADAS components were identified, which paralleled those previously seen following MTX, suggesting common outcome patterns across the JADAS components following DMARD therapy in JIA. Changing trajectory patterns following different therapies was demonstrated as possible. These outcome trajectories present a common outcome by which early stratification of DMARDs could be tailored in JIA.

## Supplementary Material

rkag088_Supplementary_Data

## Data Availability

Information regarding applying for access to the UK JIA Biologics Register, CAPS and/or CHARMS data can be found at http://www.caps-jia.org.uk/clinicians-and-researchers/, https://sites.manchester.ac.uk/bcrdbspar/ and https://www.clusterconsortium.org.uk/2349-2/.

## Cluster Co-investigators

Lucy R. Wedderburn (UCL GOS Institute of Child Health, London), Andrew Dick (UCL Institute of Ophthalmology, London), Michael W. Beresford (University of Liverpool and Alder Hey Children’s NHS Foundation Trust, Liverpool), Athimalaipet V. Ramanan (University Hospitals Bristol and University of Bristol), Michael Barnes (Queen Mary University, London), Stephen Eyre (University of Manchester), Kimme Hyrich (University of Manchester), Soumya Raychaudhuri (University of Manchester), Chris Wallace (University of Cambridge) and Nophar Geifman (University of Surrey).

## Members of the CLUSTER Consortium

Lucy R. Wedderburn, Melissa Kartawinata, Zoe Wanstall, Bethany R. Jebson, Alyssia McNeece, Elizabeth Ralph, Vasiliki Alexiou, Fatjon Dekaj, Aline Kimonyo, Fatema Merali, Emma Sumner, Emily Robinson, Andrew Dick, Michael W. Beresford, Emil Carlsson, Joanna Fairlie, Jenna F. Gritzfeld, Athimalaipet Ramanan, Teresa Duerr, Michael Barnes, Sandra Ng, Kimme Hyrich, Stephen Eyre, Soumya Raychaudhuri, Andrew Morris, Annie Yarwood, Samantha Smith, Stevie Shoop-Worrall, Saskia Lawson-Tovey, John Bowes, Paul Martin, Melissa Tordoff, Michael Stadler, Wendy Thomson, Damian Tarasek, Chris Wallace, Wei-Yu Lin, Nophar Geifman, Sarah Clarke. Victoria J. Burton, Thierry Sornasse, Daniela Dastros-Pitei, Sumanta Mukherjee, Jacqui Roberts, Rami Kallala, Helen Neale, John Ioannou and Hussein Al-Mossawi.

## The CLUSTER Champions CHARMS study group

Eslam Al-Abadi, Vicky Alexiou, Cherelle Allen, Kate Armon, Rehana Begum, Rumena Begum, Mariejennelynn Bostock, Katrin Buerkle, Charlotte Busby, Maryam Butt, Nga Sze (Emily) Cheng, Chia-Ping Chou, Joanna Cobb, Louise Coke, Julie Cook, Jenny Crook, Serena Cruickshank-Hull, Karen Davies, Lucinda Dawson, Fatjon Dekaj, Monika Dimitrova, Julie Enright, Angela Etheridge, Elizabeth (Lizzie) Fofana, Sara Foster, Sophie Foxall, Paul Gilbert, Genevieve Gottschalk, Eileen Hahn, Jeannette Hall, Daniel Hawley, Anne Hinks, Shashi Hirani, Ruth Howman, Alisha Hussein, Fatema Jeraj, Emma Jordan, Melissa Kartawinata, Laura Kassoumeri, Aline Kimonyo, Klaudia Kupiec, Sham Lal, Alice Leahy, Freya Luling Feilding, Ian MacDonald, Alyssia McNeece, Laura Melville, Fatima Merali, Halima Moncrieffe, Gudrun Moore, Kathleen Mulligan, Stanton Newman, Lucy Nguyen, Fiona Patrick, Hannah Peckham, Chadwick Pils, Elizabeth Ralph, Rachel Rikunenko, Emily Robinson, Jennie Sharp, Taunton Southwood, Jason Sowter, Mohammed Zaffar Ullah, Wendy Thomson, Simona Ursu, Hemlata Varsani, Kishore Warrier, Lucy R Wedderburn, Pamela Whitworth, Rachel Wiffen, Alexis Wormall, Patricia Woo, Brogan Wrest and patient/parent representatives.
