# Supplementary material for: A stratified approach to juvenile idiopathic arthritis: leveraging machine learning to uncover subgroups of patients following treatment with etanercept
Source: Rheumatol Adv Pract. 2026 Jul 20;10(3):rkag088. doi: 10.1093/rap/rkag088 (PMC13461192; doi:10.1093/rap/rkag088)
Supplement: Supplementary file 1 [file rkag088_supplementary_data.docx]

**SUPPLEMENTARY MATERIALS**

**Supplementary Table S1.** Baseline characteristics at ETN initiation across study population and three contributing cohorts

| **Characteristic at drug initiation** | **N (%) with available variable data** | **N (%) or Median (IQR)** | | | |
| --- | --- | --- | --- | --- | --- |
|  |  | **Whole cohort** | **JIA Biologics Registers** | **CAPS** | **CHARMS** |
| N* | - | 1003 | 787 | 214 | 37 |
| **Demographic** | | | | | |
| Female | 992 (99) | 685 (69) | 544 (69.5) | 147 (68.7) | 22 (73.3) |
| Age | 994 (99) | 12 (8, 14) | 11.9 (8.4, 14.5) | 10.1 (6.4, 13.5) | 10.6 (6.3, 12.7) |
| Disease duration (yrs) | 248 (25) | 2 (1, 4) | 2.1 (1.1, 4.0) | 2.1 (1.1, 4.1) | 3.3 (1.7, 7.8) |
| White ethnicity | 959 (96) | 864 (90) | 682 (89.4) | 190 (94.5) | 25 (83.3) |
| **ILAR category** | | | | | |
| Oligo (Persistent) | 996 (99) | 87 (9) | 52 (6.6) | 35 (16.7) | 4 (13.3) |
| Oligo (Extended) |  | 202 (20) | 174 (22.1) | 20 (10.0) | 4 (13.3) |
| RF – poly |  | 365 (37) | 291 (37.0) | 82 (39.2) | 13 (43.3) |
| RF + poly |  | 103 (10) | 91 (11.6) | 13 (6.2) | 5 (16.7) |
| ERA |  | 107 (11) | 85 (10.8) | 25 (12.0) | 1 (3.3) |
| PsA |  | 59 (6) | 49 (6.2) | 12 (5.7) | 2 (6.7) |
| Undifferentiated |  | 73 (7) | 44 (5.6) | 22 (10.5) | 1 (3.3) |
| **Clinical** | | | | | |
| Active joint count | 805 (80) | 5 (3, 9) | 5 (3, 9) | 4 (2, 7) | 4 (2, 7) |
| Limited joint count | 792 (79) | 4 (2, 8) | 4 (2, 8) | 2 (1, 5) | 3 (2, 6) |
| Physician global (cm) | 634 (63) | 3.5 (2.0, 5.0) | 3.7 (2.0, 5.4) | 2.5 (5.0, 4.9) | 4.0 (2.5, 5.0) |
| ESR (mm/hr) | 722 (72) | 12 (5, 28) | 13 (5, 29) | 10 (4, 19) | 20 (8, 35) |
| History of uveitis | 721 (72) | 50 (7) | 50 (6.9) | 2 (6.7) | 1 (33.3) |
| Parent global (cm) | 606 (60) | 4.4 (2.0, 6.6) | 4.5 (2.0, 6.8) | 3.6 (1.5, 5.3) | 4.8 (2.5, 5.7) |
| CHAQ | 518 (52) | 1.0 (0.4, 1.6) | 1.0 (0.4, 1.8) | 0.6 (0.3, 1.5) | 0.8 (0.5, 1.5) |
| Pain (cm) | 509 (51) | 5.0 (2.2, 7.0) | 5.0 (2.3, 7.0) | 3.8 (1.2, 6.7) | - |
| Concurrent MTX with ETN | 1003 (100) | 501 (50) | 351 (44.6) | 137 (64.0) | 32 (86.5) |

*n=35 children and young people contributed different follow-up data points from multiple studies

**Supplementary Table S2.** Model diagnostics across potential multi-trajectory models (adjusting for year (<2006, >=2006), n=1003)

| **Class numbers** | **AIC** | **BIC** | **Class proportions** | | | | | | | | | | **Average posterior probability per class** | | | | | | | | | | **Model average posterior probability for assigned class** | **Relative Entropy** | |
| --- | --- | --- | --- | --- | --- | --- | --- | --- | --- | --- | --- | --- | --- | --- | --- | --- | --- | --- | --- | --- | --- | --- | --- | --- | --- |
|  |  |  | **1** | **2** | **3** | **4** | **5** | **6** | **7** | **8** | **9** | **10** | **1** | **2** | **3** | **4** | **5** | **6** | **7** | **8** | **9** | **10** |  |  |  |
| **Linear** | | | | | | | | | | | | | | | | | | | | | | | | | |
| **1** | -14770 | -14799 | 1 |  |  |  |  |  |  |  |  |  | 1 |  |  |  |  |  |  |  |  |  | 1 | 1 |  |
| **2** | -13995 | -14049 | 0.63 | 0.37 |  |  |  |  |  |  |  |  | 0.94 | 0.93 |  |  |  |  |  |  |  |  | 0.93 | 0.89 |  |
| **3** | -13788 | -13866 | 0.41 | 0.35 | 0.25 |  |  |  |  |  |  |  | 0.87 | 0.83 | 0.88 |  |  |  |  |  |  |  | 0.86 | 0.76 |  |
| **4** | -13692 | -13795 | 0.44 | 0.27 | 0.21 | 0.08 |  |  |  |  |  |  | 0.88 | 0.80 | 0.87 | 0.83 |  |  |  |  |  |  | 0.85 | 0.74 |  |
| **5** | -13597 | -13724 | 0.43 | 0.26 | 0.13 | 0.11 | 0.07 |  |  |  |  |  | 0.87 | 0.80 | 0.86 | 0.79 | 0.85 |  |  |  |  |  | 0.83 | 0.71 |  |
| **6** | -13539 | -13691 | 0.30 | 0.27 | 0.19 | 0.11 | 0.09 | 0.04 |  |  |  |  | 0.81 | 0.79 | 0.80 | 0.85 | 0.81 | 0.86 |  |  |  |  | 0.82 | 0.67 |  |
| **7** | -13477 | -13654 | 0.32 | 0.26 | 0.11 | 0.11 | 0.10 | 0.05 | 0.03 |  |  |  | 0.82 | 0.78 | 0.81 | 0.77 | 0.86 | 0.86 | 0.82 |  |  |  | 0.82 | 0.65 |  |
| **8** | -13398 | -13599 | 0.24 | 0.23 | 0.15 | 0.10 | 0.09 | 0.09 | 0.06 | 0.04 |  |  | 0.78 | 0.71 | 0.72 | 0.82 | 0.85 | 0.75 | 0.79 | 0.85 |  |  | 0.78 | 0.57 |  |
| **9** | -13363 | -13589 | 0.25 | 0.17 | 0.16 | 0.10 | 0.09 | 0.08 | 0.07 | 0.04 | 0.03 |  | 0.77 | 0.70 | 0.73 | 0.81 | 0.85 | 0.77 | 0.73 | 0.87 | 0.85 |  | 0.79 | 0.56 |  |
| **10*** | -13311 | -13562 | 0.24 | 0.17 | 0.10 | 0.10 | 0.10 | 0.09 | 0.07 | 0.06 | 0.04 | 0.03 | 0.77 | 0.71 | 0.79 | 0.74 | 0.73 | 0.85 | 0.78 | 0.76 | 0.87 | 0.86 | 0.78 | 0.56 |  |
| **Quadratic** | | | | | | | | | | | | | | | | | | | | | | | | | |
| **1** | -14525 | -14564 | 1 |  |  |  |  |  |  |  |  |  | 1 |  |  |  |  |  |  |  |  |  | 1 | 1 |  |
| **2** | -13776 | -13850 | 0.59 | 0.41 |  |  |  |  |  |  |  |  | 0.93 | 0.92 |  |  |  |  |  |  |  |  | 0.92 | 0.88 |  |
| **3** | -13571 | -13679 | 0.40 | 0.38 | 0.23 |  |  |  |  |  |  |  | 0.84 | 0.87 | 0.89 |  |  |  |  |  |  |  | 0.87 | 0.77 |  |
| **4** | -13484 | -13627 | 0.39 | 0.35 | 0.19 | 0.07 |  |  |  |  |  |  | 0.88 | 0.82 | 0.89 | 0.85 |  |  |  |  |  |  | 0.85961 | 0.742 |  |
| **5** | -13404 | -13580 | 0.38 | 0.30 | 0.16 | 0.09 | 0.06 |  |  |  |  |  | 0.86 | 0.79 | 0.85 | 0.76 | 0.85 |  |  |  |  |  | 0.82 | 0.69 |  |
| **6*** | -13356 | -13567 | 0.34 | 0.28 | 0.20 | 0.09 | 0.05 | 0.03 |  |  |  |  | 0.85 | 0.80 | 0.81 | 0.85 | 0.85 | 0.81 |  |  |  |  | 0.83 | 0.68 |  |
| **7*** | -13268 | -13514 | 0.35 | 0.27 | 0.10 | 0.09 | 0.08 | 0.06 | 0.06 |  |  |  | 0.84 | 0.78 | 0.80 | 0.81 | 0.77 | 0.84 | 0.82 |  |  |  | 0.81 | 0.65 |  |
| **8*** | -13210 | -13490 | 0.32 | 0.27 | 0.11 | 0.10 | 0.09 | 0.05 | 0.04 | 0.03 |  |  | 0.83 | 0.73 | 0.79 | 0.80 | 0.78 | 0.83 | 0.82 | 0.89 |  |  | 0.81 | 0.62 |  |
| **9*** | -13197 | -13512 | 0.31 | 0.26 | 0.10 | 0.09 | 0.08 | 0.06 | 0.04 | 0.04 | 0.01 |  | 0.80 | 0.74 | 0.79 | 0.82 | 0.77 | 0.83 | 0.81 | 0.88 | 0.88 |  | 0.81 | 0.61 |  |
| **10** | -13180 | -13528 | 0.33 | 0.24 | 0.09 | 0.09 | 0.07 | 0.06 | 0.05 | 0.03 | 0.03 | 0.02 | 0.83 | 0.77 | 0.75 | 0.75 | 0.83 | 0.82 | 0.86 | 0.82 | 0.80 | 0.80 | 0.79 | 0.61 |  |
| **Cubic** | | | | | | | | | | | | | | | | | | | | | | | | | |
| **1** | -14487 | -145356 | 1 |  |  |  |  |  |  |  |  |  | 1 |  |  |  |  |  |  |  |  |  | 1 | 1 |  |
| **2** | -13713 | -13806 | 0.59 | 0.41 |  |  |  |  |  |  |  |  | 0.93 | 0.93 |  |  |  |  |  |  |  |  | 0.93 | 0.88 |  |
| **3** | -13515 | -13652 | 0.40 | 0.36 | 0.24 |  |  |  |  |  |  |  | 0.84 | 0.88 | 0.89 |  |  |  |  |  |  |  | 0.87 | 0.78 |  |
| **4** | -13426 | -13608 | 0.41 | 0.31 | 0.20 | 0.08 |  |  |  |  |  |  | 0.88 | 0.82 | 0.85 | 0.83 |  |  |  |  |  |  | 0.84 | 0.74 |  |
| **5** | -13316 | -13542 | 0.34 | 0.29 | 0.15 | 0.13 | 0.09 |  |  |  |  |  | 0.85 | 0.77 | 0.79 | 0.80 | 0.83 |  |  |  |  |  | 0.81 | 0.67 |  |
| **6*** | -13242 | -13512 | 0.33 | 0.29 | 0.12 | 0.11 | 0.11 | 0.04 |  |  |  |  | 0.84 | 0.76 | 0.82 | 0.82 | 0.83 | 0.85 |  |  |  |  | 0.82 | 0.67 |  |
| **7*** | -13144 | -13459 | 0.32 | 0.29 | 0.10 | 0.10 | 0.08 | 0.07 | 0.04 |  |  |  | 0.84 | 0.77 | 0.83 | 0.83 | 0.82 | 0.90 | 0.82 |  |  |  | 0.83 | 0.67 |  |
| **8*** | -13131 | -13490 | 0.32 | 0.21 | 0.13 | 0.10 | 0.07 | 0.07 | 0.06 | 0.04 |  |  | 0.84 | 0.74 | 0.83 | 0.73 | 0.82 | 0.77 | 0.80 | 0.82 |  |  | 0.79 | 0.61 |  |
| **9*** | -13095 | -13498 | 0.31 | 0.27 | 0.10 | 0.08 | 0.07 | 0.07 | 0.04 | 0.03 | 0.02 |  | 0.84 | 0.78 | 0.81 | 0.82 | 0.81 | 0.85 | 0.88 | 0.92 | 0.84 |  | 0.84 | 0.66 |  |
| **10*** | -13022 | -13468 | 0.30 | 0.25 | 0.09 | 0.08 | 0.07 | 0.06 | 0.06 | 0.05 | 0.03 | 0.03 | 0.82 | 0.76 | 0.82 | 0.87 | 0.76 | 0.78 | 0.83 | 0.83 | 0.82 | 0.85 | 0.82 | 0.64 |  |

a)
b)
c)

**Supplementary Figure S1.** Model fit and proportion in each class across multivariate trajectory models of JADAS components following ETN, adjusting for year of ETN initiation (<2006, >=2006): a. Linear, b. Quadratic, c. Cubic. *Unstable estimates or non-convergence

**Supplementary Figure S2.** Average raw active joint count, physician global, patient/parent global and ESR trajectories within five multivariate disease clusters over the year following ETN initiation. Shaded areas represent 95% confidence intervals around fitted mean trajectories.

a)

b)

**Supplementary Figure S3.** Time to a) ETN stop and b) second biologic switch across the trajectory clusters

**Supplementary Table S3.** Univariable associations between characteristics at ETN initiation and ETN response trajectory compared with Persistent Disease following multiple imputation

| **Baseline variable** | **N (%) or Median (IQR)** | | | | |
| --- | --- | --- | --- | --- | --- |
|  | **Group 1:**  **Persistent PGA** | **Group 2:**  **Fast improvers** | **Group 3:**  **Persistent PGE** | **Group 4:**  **Persistent disease** | **Group 5:**  **Slow Improvers** |
| **N (%)** | 64 (6) | 160 (16) | 303 (30) | 383 (38) | 93 (9) |
| **Demographic** |  |  |  |  |  |
| Age at registration (years) | 0.99 (0.92, 1.05) | 0.97 (0.92, 1.01) | 1.04 (1.00, 1.07) | Reference | 1.02 (0.96, 1.08) |
| Disease duration to registration (~ETN initiation) (years) | 1.05 (0.63, 1.75) | 0.98 (0.49, 1.96) | 1.04 (0.74, 1.46) | Reference | 1.14 (1.00, 1.29) |
| Female | 1.13 (0.63, 2.04) | 1.02 (0.67, 1.54) | **1.47 (1.06, 2.03)** | Reference | 1.08 (0.65, 1.78) |
| White ethnicity | **8.61 (1.17, 64.41)** | 0.96 (0.54, 1.71) | **2.08 (1.19, 3.62)** | Reference | 1.74 (0.75, 4.01) |
| **ILAR category** |  |  |  |  |  |
| Systemic | - | - | **-** | - | - |
| Oligo – Persistent | Reference | Reference | Reference | Reference | Reference |
| Oligo – Extended | **0.33 (0.11, 0.97)** | **0.21 (0.10, 0.45)** | **0.32 (0.16, 0.63)** | Reference | 2.50 (0.54, 11.67) |
| RF- Poly | 0.39 (0.14, 1.05) | **0.21 (0.10, 0.43)** | **0.30 (0.16, 0.57)** | Reference | 1.47 (0.32, 6.78) |
| RF+ Poly | **0.17 (0.04, 0.68)** | **0.24 (0.11, 0.56)** | **0.17 (0.08, 0.38)** | Reference | 1.38 (0.27, 7.09) |
| Psoriatic | 0.32 (0.09, 1.16) | **0.28 (0.12, 0.68)** | 0.49 (0.23, 1.04) | Reference | 2.43 (0.48, 12.32) |
| Enthesitis-related | 0.24 (0.05, 1.06) | **0.16 (0.05, 0.44)** | **0.24 (0.10, 0.57)** | Reference | 1.67 (0.30, 9.31) |
| Undifferentiated | 0.43 (0.11, 1.60) | **0.29 (0.11, 0.74)** | **0.23 (0.09, 0.55)** | Reference | 2.40 (0.45, 12.84) |
| **Disease activity** |  |  |  |  |  |
| Active joint count | 0.97 (0.81, 1.16) | 0.97 (0.89, 1.07) | 0.97 (0.91, 1.03) | Reference | **0.96 (0.93, 1.00)** |
| Limited joint count  (excluded from multivariable model due to collinearity with AJC) | 1.00 (0.90, 1.10) | 0.98 (0.90, 1.06) | 0.96 (0.89, 1.04) | Reference | 0.98 (0.95, 1.01) |
| Physician’s global (mm) | 0.98 (0.94, 1.02) | 0.99 (0.95, 1.02) | 0.98 (0.97, 1.00) | Reference | **0.99 (0.97, 1.00)** |
| Parental global (mm) | 0.94 (0.85, 1.03) | 0.98 (0.95, 1.02) | 0.99 (0.97, 1.01) | Reference | **0.98 (0.97, 0.99)** |
| CHAQ | 0.20 (0.02, 2.07) | 0.43 (0.08, 2.31) | 0.53 (0.11, 2.63) | Reference | **0.43 (0.29, 0.64)** |
| ESR | 0.97 (0.93, 1.01) | 0.99 (0.98, 1.01) | **0.95 (0.91, 0.99)** | Reference | 1.00 (0.99, 1.01) |
| Concurrent MTX | 0.99 (0.58, 1.69) | 0.89 (0.61, 1.29) | 0.95 (0.70, 1.29) | Reference | 1.42 (0.90, 2.26) |
| Uveitis history | 0.98 (0.22, 4.47) | 1.24 (0.50, 3.09) | 1.61 (0.80, 3.23) | Reference | 1.99 (0.74, 5.17) |

**Supplementary Table S4.** Multivariable associations between characteristics at ETN initiation and ETN disease impact trajectory compared with Persistent Disease following multiple imputation

| **Baseline variable** | **RRR (95% CI)** | | | | |
| --- | --- | --- | --- | --- | --- |
|  | **Group 1:**  **Persistent PGA** | **Group 2:**  **Fast improvers** | **Group 3:**  **Persistent PGE** | **Group 4:**  **Persistent disease** | **Group 5:**  **Slow Improvers** |
| **N (%)** | 64 (6) | 160 (16) | 303 (30) | 383 (38) | 93 (9) |
| **Demographic** |  |  |  |  |  |
| Age at registration (years) | 0.94 (0.84, 1.05) | **0.91 (0.84, 0.98)** | 1.02 (0.96, 1.07) | Reference | 0.99 (0.91, 1.07) |
| Disease duration to registration (~ETN initiation) (years) | 1.10 (0.90, 1.36) | 1.10 (0.90, 1.35) | 0.99 (0.87, 1.14) | Reference | 1.12 (0.93, 1.34) |
| Female | 1.23 (0.58, 2.59) | 1.01 (0.61, 1.67) | 1.14 (0.75, 1.74) | Reference | 1.06 (0.60, 1.89) |
| White ethnicity | 6.30 (0.71, 55.85) | 1.01 (0.51, 1.99) | 1.59 (0.85, 2.96) | Reference | 1.73 (0.71, 4.21) |
| **ILAR category** |  |  |  |  |  |
| Systemic | - | - | **-** | - | - |
| Oligo – Persistent | Reference | Reference | Reference | Reference | Reference |
| Oligo – Extended | 0.71 (0.21, 2.41) | **0.25 (0.10, 0.66)** | 0.58 (0.26, 1.29) | Reference | 3.33 (0.65, 16.91) |
| RF- Poly | 1.45 (0.44, 4.79) | **0.40 (0.17, 0.96)** | 0.58 (0.27, 1.24) | Reference | 3.02 (0.61, 14.97) |
| RF+ Poly | 0.51 (0.10, 2.54) | 0.54 (0.19, 1.51) | **0.35 (0.14, 0.69)** | Reference | 2.36 (0.43, 12.80) |
| Psoriatic | 1.11 (0.21, 5.80) | 0.69 (0.23, 1.98) | 0.82 (0.33, 2.01) | Reference | 4.17 (0.76, 22.81) |
| Enthesitis-related | 0.96 (0.15, 6.15) | 0.43 (0.12, 1.54) | 0.39 (0.15, 1.06) | Reference | 3.73 (0.58, 23.97) |
| Undifferentiated | 1.29 (0.26, 6.54) | 0.61 (0.19, 2.01) | 0.49 (0.18, 1.34) | Reference | 4.94 (0.81, 29.94) |
| **Disease activity** |  |  |  |  |  |
| Active joint count | 0.96 (0.90rt, 1. N89im03) | 0.98 (0.94, 1.02) | 0.98 (0.95, 1.00) | Reference | 0.97 (0.91, 1.03) |
| Limited joint count  (excluded due to collinearity with AJC) | - | - | - | Reference | - |
| Physician’s global (cm) | 0.99 (0.81, 1.21) | 0.99 (0.86, 1.14) | 0.95 (0.85, 1.06) | Reference | 1.01 (0.86, 1.19) |
| Parental global (cm) | **0.57 (0.41, 0.79)** | 1.04 (0.87, 1.24) | 1.00 (0.90, 1.12) | Reference | 0.88 (0.73, 1.05) |
| CHAQ | **0.20 (0.07, 0.57)** | **0.37 (0.22, 0.64)** | **0.68 (0.48, 0.97)** | Reference | **0.54 (0.32, 0.90)** |
| ESR | 0.98 (0.95, 1.01) | 1.00 (0.99, 1.01) | **0.94 (0.92, 0.96)** | Reference | 1.00 (1.00, 1.01) |
| Pain (cm) | 1.02 (0.98, 1.08) | 0.88 (0.73, 1.05) | 1.00 (0.98, 1.03) | Reference | 0.96 (0.85, 1.09) |
| Concurrent MTX | 1.49 (0.76, 2.96) | 0.93 (0.59, 1.49) | 1.08 (0.75, 1.55) | Reference | **1.77 (1.04, 3.01)** |

Bolded values are statistically significant (p<0.05) under multivariable multinomial logistic regression

**Supplementary Table S5.** Multivariable associations between characteristics at ETN initiation and each ETN response trajectory (yes/no) following multiple imputation

| **Baseline variable** | **RRR (95% CI)** | | | | |
| --- | --- | --- | --- | --- | --- |
|  | **Fast improvers** | **Slow Improvers** | **Persistent PGA** | **Persistent PGE** | **Persistent disease** |
| **N (%)** | 160 (16) | 93 (9) | 64 (6) | 303 (30) | 383 (38) |
| **Demographic** |  |  |  |  |  |
| Age at registration (years) | **0.90 (0.85, 0.96)** | 0.98 (0.94, 1.02) | 0.96 (0.87, 1.06) | **1.05 (1.00, 1.10)** | 1.02 (0.97, 1.07) |
| Disease duration to registration (~ETN initiation) (years) | 1.07 (0.92, 1.25) | 1.08 (0.91, 1.28) | 1.05 (0.88, 1.26) | 0.94 (0.84, 1.06) | 0.95 (0.84, 1.07) |
| Female | 0.91 (0.59, 1.42) | 0.97 (0.56, 1.69) | 1.09 (0.53, 2.23) | 1.09 (0.76, 1.59) | 0.88 (0.62, 1.26) |
| White ethnicity | 0.71 (0.39, 1.30) | 1.39 (0.59, 3.28) | 5.18 (0.65, 41.22) | 1.37 (0.76, 2.46) | 0.71 (0.43, 1.16) |
| **ILAR category** |  |  |  |  |  |
| Systemic | - | - | - | **-** | - |
| Oligo – Persistent | Reference | Reference | Reference | Reference | Reference |
| Oligo – Extended | **0.39 (0.19, 0.81)** | **6.62 (1.44, 30.36)** | 1.19 (0.38, 3.70) | 0.84 (0.44, 1.60) | 1.92 (0.94, 3.92) |
| RF- Poly | 0.55 (0.30, 1.04) | **4.89 (1.09, 21.94)** | 2.07 (0.71, 5.99) | 0.72 (0.40, 1.28) | 1.63 (0.81, 3.29) |
| RF+ Poly | 1.01 (0.45, 2.28) | 4.67 (0.91, 24.03) | 0.86 (0.19, 3.89) | **0.45 (0.21, 0.97)** | 2.23 (0.98, 5.05) |
| Psoriatic | 0.79 (0.34, 1.84) | **5.82 (1.14, 29.72)** | 1.23 (0.29, 5.27) | 0.90 (0.45, 1.81) | 1.24 (0.53, 2.91) |
| Enthesitis-related | 0.64 (0.23, 1.80) | **7.01 (1.21, 40.72)** | 1.66 (0.30, 9.09) | 0.49 (0.21, 1.15) | 2.03 (0.82, 5.04) |
| Undifferentiated | 0.84 (0.34, 2.06) | **7.89 (1.47, 42.20)** | 1.65 (0.39, 6.95) | 0.52 (0.22, 1.19) | 1.42 (0.58, 3.50) |
| **Disease activity** |  |  |  |  |  |
| Active joint count | 0.99 (0.96, 1.03) | 0.98 (0.94, 1.02) | 0.97 (0.91, 1.04) | 0.99 (0.96, 1.01) | **1.02 (1.00, 1.05)** |
| Limited joint count  (excluded from multivariable model due to collinearity with AJC) | - | - | - | - | - |
| Physician’s global (cm) | 1.00 (0.89, 1.14) | 1.03 (0.88, 1.20) | 1.02 (0.83, 1.25) | 0.96 (0.85, 1.07) | 1.03 (0.95, 1.13) |
| Parental global (cm) | 1.01 (0.89, 1.14) | 0.89 (0.77, 1.02) | **0.61 (0.47, 0.80)** | 1.09 (0.99, 1.19) | 1.07 (0.97, 1.17) |
| Pain (cm) (excluded from multivariable model due to collinearity with parental global) |  |  |  |  |  |
| CHAQ | **0.46 (0.29, 0.73)** | 0.79 (0.49, 1.26) | **0.32 (0.12, 0.86)** | 1.05 (0.77, 1.41) | **1.97 (1.41, 2.75)** |
| ESR | **1.01 (1.00, 1.02)** | **1.01 (1.00, 1.02)** | 0.99 (0.97, 1.01) | **0.94 (0.92, 0.96)** | 1.02 (1.01, 1.02) |
| Concurrent MTX | 0.80 (0.54, 1.18) | **1.68 (1.05, 2.70)** | 1.34 (0.72, 2.51) | 1.00 (0.73, 1.38) | 0.85 (0.62, 1.16) |

**Supplementary Table S6.** Number and percentage of those in each MTX trajectory assigned to each ETN trajectory

| MTX Trajectory | N(%) of those in each MTX Trajectory who were assigned to each ETN Trajectory | | | | |
| --- | --- | --- | --- | --- | --- |
|  | Fast Improvers | Slow Improvers | Persistent PGA | Persistent PGE | Persistent Disease |
| Fast Improvers | 4 (50.0) | 0 (0.0) | 1 (12.5) | 2 (25.0) | 1 (12.5) |
| Slow Improvers | 3 (21.4) | 1 (7.1) | 1 (7.1) | 4 (28.6) | 5 (35.7) |
| Persistent PGA | 3 (18.8) | 0 (0.0) | 4 (25.0) | 4 (25.0) | 5 (31.3) |
| Persistent PGE | 2 (13.3) | 1 (6.7) | 1 (6.7) | 11 (73.3) | 0 (0.0) |
| Persistent Disease | 7 (8.1) | 13 (15.1) | 9 (10.5) | 30 (34.9) | 27 (31.4) |

Code excerpt

An example of code implemented in STATA 14 for multivariate group-based trajectory modelling, adjusting for year of ETN start (<2006, 2006+) is given. The following will build a 5-class multitrajectory model with linear-only, quadratic-only and cubic-only polynomials within each loop. This was repeated, varying the number of classes from 1-10. Data are in wide format, with one variable per outcome for each month of follow-up (e.g. activejoints0 activejoints1 activejoints2 etc).

*log1p transform outcome vars

foreach i of numlist 0/14 {

foreach var in activejoints physicianglobal patientparentglobal esr {

gen log_`var'`i'=log(`var'`i'+1)

}

}

*Run a 5-group multitrajectory model, adjusting for year of ETN start (variable: year_start; values: 0=<2006,1= ≥2006).

foreach i in 1 2 3 {

traj, multgroups(5) ///

multrisk(year_start) ///

var(log_activejoints*) ///

indep(has_fup*) ///

model(cnorm) ///

order(`i' `i' `i' `i' `i') ///

min(0) max(6) ///

var2(log_physicianglobal*) ///

indep2(has_fup*) ///

model2(cnorm) ///

order2(`i' `i' `i' `i' `i') ///

min2(0) max2(6) ///

var3(log_patientparentglobal*) ///

indep3(has_fup*) ///

model3(cnorm) ///

order3(`i' `i' `i' `i' `i') ///

min3(0) max3(6) ///

var4(log_esr*) ///

indep4(has_fup*) ///

model4(cnorm) ///

order4(`i' `i' `i' `i' `i') ///

min4(0) max4(6)

}

*Tabulate numbers in each assigned cluster

tab _traj_Group

* Summarise mean posterior probabilities of assigned cluster

foreach i in 1 2 3 4 5 {

sum _traj_ProbG`i' if _traj_Group==`i'

}
